# Computational Saturation Mutagenesis of SARS-CoV-1 Spike Glycoprotein: Stability, Binding Affinity, and Comparison With SARS-CoV-2

**DOI:** 10.3389/fmolb.2021.784303

**Published:** 2021-12-09

**Authors:** Adebiyi Sobitan, Vidhyanand Mahase, Raina Rhoades, Dejaun Williams, Dongxiao Liu, Yixin Xie, Lin Li, Qiyi Tang, Shaolei Teng

**Affiliations:** ^1^ Department of Biology, Howard University, Washington, DC, United States; ^2^ Howard University College of Medicine, Washington, DC, United States; ^3^ Computational Science Program, University of Texas at El Paso, El Paso, TX, United States; ^4^ Physics Department, University of Texas at El Paso, El Paso, TX, United States

**Keywords:** computational saturation mutagenesis, spike missense mutations, SARS-CoV-1, SARS-CoV-2, protein stability, binding affinity

## Abstract

Severe Acute respiratory syndrome coronavirus (SARS-CoV-1) attaches to the host cell surface to initiate the interaction between the receptor-binding domain (RBD) of its spike glycoprotein (S) and the human Angiotensin-converting enzyme (hACE2) receptor. SARS-CoV-1 mutates frequently because of its RNA genome, which challenges the antiviral development. Here, we per-formed computational saturation mutagenesis of the S protein of SARS-CoV-1 to identify the residues crucial for its functions. We used the structure-based energy calculations to analyze the effects of the missense mutations on the SARS-CoV-1 S stability and the binding affinity with hACE2. The sequence and structure alignment showed similarities between the S proteins of SARS-CoV-1 and SARS-CoV-2. Interestingly, we found that target mutations of S protein amino acids generate similar effects on their stabilities between SARS-CoV-1 and SARS-CoV-2. For example, G839W of SARS-CoV-1 corresponds to G857W of SARS-CoV-2, which decrease the stability of their S glycoproteins. The viral mutation analysis of the two different SARS-CoV-1 isolates showed that mutations, T487S and L472P, weakened the S-hACE2 binding of the 2003–2004 SARS-CoV-1 isolate. In addition, the mutations of L472P and F360S destabilized the 2003–2004 viral isolate. We further predicted that many mutations on N-linked glycosylation sites would increase the stability of the S glycoprotein. Our results can be of therapeutic importance in the design of antivirals or vaccines against SARS-CoV-1 and SARS-CoV-2.

## Introduction

The severe acute respiratory syndrome coronavirus (SARS-CoV-1) belongs to a family of Coronaviridae that are enveloped, positive-strand RNA viruses (Li F. et al., 2005). In November 2002, the first case of SARS-CoV-1 occurred in the Guangdong province in China. The symptoms of SARS include upper respiratory infections, fever, chills, and general body weakness (Peiris et al., 2003). The other signs showing human-to-human transmission were coughing and sneezing. Horseshoe bat species might be the origin due to their sequence similarity (Luk et al., 2019). By the end of the SARS epidemic in 2003, SARS had spread to over two dozen countries resulting in more than 8,000 laboratory-confirmed cases and approximately 800 deaths (www.cdc.gov) (Centers for Disease Control and Prevention, 2021). However, in 2004, there were four mild cases of SARS-CoV-1 outbreak. The recent outbreak of a newer strain of coronavirus, SARS-CoV-2, began in December 2019 in the Wuhan city in China (Vankadari and Wilce, 2020). In a month, this new coronavirus had spread across the world due to global travels. Compared to SARS-CoV-1, SARS-CoV-2 has a higher infection rate. As of November 11, 2021, the number of confirmed global cases and global deaths due to SARS-CoV-2 are ∼252 million and ∼5.1 million, respectively (Dong et al., 2020).

The Spike protein (S) is a structural protein that protrudes outwards from the virus surface. The role of S is to mediate viral entry into the host’s cells. Structural studies of SARS-CoV-1’s S revealed the presence of two subunits, S1 (residues 12–667) is in the N-terminal and S2 (residues 667–1,190) is in the C-terminal. Studies of mammalian coronaviruses with similarity to the SARS-CoV-1 showed that the S1 subunit helps with hACE2 receptor attachment, while the S2 subunit helps with the fusion (Li F. et al., 2005). Expression analysis showed that a fragment of the S1 subunit, the receptor binding domain (RBD), residues 306–527, is enough for tight binding to the human Angiotensin-converting enzyme 2 (hACE2) receptor (Song et al., 2018). A shorter fragment, residues 424–494, within the RBD interacts with the hACE2 receptor in humans. This fragment, the receptor-binding motif (RBM), forms a loop that fits perfectly into the peptidase do-main (PD) of the hACE2 receptor. The SARS-CoV-1 glycoprotein has two cleavage sites that promote viral infection. The first cleavage site is in the 667–668 residue positions. As the virus enters the host’s cell, its spike protein is cleaved into the S1 and S2 subunit by protease activity. The second cleavage site is in the 797–798 residue positions. Cleavage at this position detaches the fusion protein from the S2 subunit and allows the fusion protein to bind with the host’s membrane (Belouzard et al., 2009).

One similarity shared by SARS-CoV-1 and SARS-CoV-2 is that they use hACE2 as the receptor to enter human cells (Manning et al., 2002). Both SARS-CoV-1 and SARS-CoV-2 S proteins bind to the peptidase domain in the N-terminal of the hACE2 receptor (Li F. et al., 2005). The other domain, the Collectrin domain, is found in the C-terminal of the hACE2 receptor. However, studies showed they exhibit varying binding affinities. A recent study reported that SARS-CoV-2 S protein has a 10-to-20-fold higher affinity to hACE2 than that of SARS-CoV-1 (Song et al., 2018). The interaction between the S protein of SARS-CoV-1 and the hACE2 receptor initiates entry into the human cell (Xie et al., 2020). The higher affinity SARS-CoV-2 has for hACE2 may explain the virulent nature of its infection (Chan et al., 2020). Another similarity is the sequence and structural homology between the S proteins of SARS-CoV-1 and SARS-CoV-2. However, despite the similarities, a study evaluated the binding of SARS-CoV-2 to experimentally verified monoclonal antibodies (mAbs) against SARS-CoV-1. The result showed a slight contrast in cross-reactivity, which had no binding between SARS-CoV-2 and the three mAbs (Wrapp et al., 2020). This result supports the hypothesis that the slight difference in their sequences/structures might be re-sponsible for the varying infectivity between SARS-CoV-1 and SARS-CoV-2. In a previous study, we indicated that SARS-CoV-2 has a stronger affinity towards hACE2 than SARS-CoV-1 because of its higher electric field density (Xie et al., 2020). The hACE2 plays a role in the renin-angiotensin pathway, that it maintains cardiovascular homeostasis (Wang et al., 2016). The hACE2 participates in microbial infection by serving as an entry point for coronaviruses (Li et al., 2003). The variation of hACE2 across species explains why SARS-CoV-1 infects humans and not rats nor mice. A study manipulated the protein sequences of the hACE2 of rats and mice by mutating specific residues to the residues in humans. The result found an increase in infectivity when the mouse or rat hACE2 has human residues in certain positions (W. Li et al., 2004). Understanding the interactions between the contact residues of SARS-CoV-1 and hACE2 can provide insights into how SARS-CoV-2 enters human cells.

SARS-CoV-1 has an unstable RNA genome, an attribute common to RNA viruses (Eigen, 1993). Our goal is to investigate the effect of all possible mutations on the functions of the SARS-CoV-1 S protein. Unlike experimental analysis, computational analysis has proven an effective method in studying protein dynamics (Teng et al., 2010). Previous studies showed the high performance of specific or general computational prediction algorithms to prioritize cancer driving mutations (Zhao et al., 2018; Chen et al., 2020). Another study identified that Foldx is a better protein engineering tool in predicting protein mutations than random based approaches (Buß et al., 2018). In our recent publication, we used a computational approach to predict and analyze missense mutations in the SARS-CoV-2 S protein (Teng et al., 2020). We predicted several missense mutations that affect the stability and binding affinity of SARS-CoV-2. We identified some target mutations D614G, N501Y, and K417N in the South Africa, United Kingdom, and Brazil variants, respectively (Teng et al., 2020). In addition, we compared the effects of mutations on stability in the closed state and open state of SARS-CoV-2 S, and the Foldx results for folding energy changes introduced by mutations are highly correlated. As a result, we employed saturated computational mutagenesis to analyze the effects of missense mutations on the stability and protein-protein interactions of SARS-CoV-1. This approach is fast and effective in identifying key residues, which will help design therapeutic drugs against SARS-CoV-1. Our results will also serve as a template to study and tackle future SARS outbreaks.

## Materials and Methods

### Structural Preparation SARS-CoV-1 and SARS-CoV-2

We obtained the 3-dimensional structures of full-length S and RBD-hACE2 of both SARS-CoV-1 and SARS-CoV-2 from the RCSB Protein Data Bank (PDB) (Protein Data Bank 2019). The structure of a trypsin-cleaved SARS-CoV-1’s spike glycoprotein (PDB ID: 6ACG) was used for stability analysis. The structure of the complex of SARS-CoV-1 S RBD and the human hACE2 receptor (PDB ID: 2AJF) was used for stability and interaction analysis. For SARS-CoV-2, we obtained the protein complex structure of RBD-hACE2 (PDB ID: 6LZG) for stability and interaction analysis. The SARS-CoV-2 S (PDB ID: 6VYB) was used only for stability analysis. For structural alignment of RBD of SARS-CoV-1 and SARS-CoV-2, we used the structures PDB ID:2AJF and PDB ID:6M17, respectively. For structural alignment of the S protein of SARS-CoV-1 and SARS-CoV-2, we used the structures PDB ID:6ACG and PDB ID:6VYB, respectively. PyMOL (Schrödinger, LLC 2015) was used for the visualization of the PDB structures and for structural alignments.

### Computational Mutagenesis and Energy Calculations

Foldx version 5 (Schymkowitz et al., 2005), coded by the Foldx Consortium, was used for mutational analysis. We used the command line interface of Foldx to mutate each residue to the other 19 residues. Foldx calculates free energy, ΔG, by using the contributions of hydrophobic and polar groups to the solvation energy, Van der Waals, hydrogen bonding, and electrostatic interactions. These energy parameters were experimentally derived (Schymkowitz et al., 2005). In this study, we used the default parameters for the computation of the wildtype and mutant free energies. The initial step used the ‘RepairPdb’ command to repair the wildtype protein structure. The ‘RepairPdb’ command finds the minimum energy conformation for the protein structure by flipping side chains of all residues, especially Asparagine, Glutamine, and Histidine to reduce steric clashes (Schymkowitz et al., 2005). This was followed by either the use of the ‘BuildModel’ command and the ‘AnalyseComplex’ command to calculate the folding energy change and the interaction or binding energy change, respectively. For each mutation, we used Foldx to calculate the folding energy change (ΔΔG) and binding energy change (ΔΔΔG) (FOLDX, 2021). The mathematical equation for the calculation of folding energy change (ΔΔG) is:

$$\Delta \Delta {\text{G}}_{\left(\text{stability}\right)}=\Delta {\text{G}}_{\left(\text{folding}\right)}\text{MUT}-\Delta {\text{G}}_{\left(\text{folding}\right)}\text{WT}$$

Theoretically, a negative ΔΔG means that the mutation leads to a more stable protein structure and a positive ΔΔG means that the mutation leads to a less stable protein structure. The five categories of the impact of the folding energy change (ΔΔG) are-highly stabilizing (ΔΔG < –2.0 kcal/mol), moderately stabilizing (–2.0 < ΔΔG < –0.5 kcal/mol), neutral (0.5 < ΔΔG < +0.5 kcal/mol), moderately destabilizing (+0.5 < ΔΔG < 2.0 kcal/mol), and highly destabilizing (ΔΔG >2.0 kcal/mol). The mathematical equation for the calculation of binding energy changes (ΔΔΔG) is:

$$\Delta \Delta \Delta {\text{G}}_{\left(\text{binding}\right)}=\Delta \Delta {\text{G}}_{\left(\text{binding}\right)}\text{MUT}-\Delta \Delta {\text{G}}_{\left(\text{binding}\right)}\text{WT}$$

A negative ΔΔΔG means the mutation strengthens the binding energy and a positive ΔΔΔG means the mutation weakens the binding energy. The effect of the binding energy changes was also classified into five categories: large affinity decrease (ΔΔΔG >0.5 kcal/mol), moderate affinity decrease (0.1 < ΔΔΔG ≤0.5), neutral (–0.1 < ΔΔΔG ≤0.1 kcal/mol), moderate affinity increase (–0.5 < ΔΔΔG ≤ –0.5 kcal/mol), and large affinity in-crease (ΔΔΔG < –0.5 kcal/mol).

### Mutation Pathogenicity and Sequence-Based Analysis

We used the Polymorphism Phenotyping v2 (PolyPhen2) (I. A. Adzhubei et al., 2010) and Screening for non-acceptable polymorphisms (SNAP) (Bromberg and Rost 2007) prediction tools to predict the pathogenicity of each missense mutations. We utilized the R programming language (https://www.r-project.org/) for data visualization for the purpose of drawing inferences. Specifically, we constructed boxplots to compare the prediction of pathogenicity between PolyPhen2 and SNAP.

### Sequence and Structural Similarity Between SARS-CoV-1 and SARS-CoV-2

The FASTA sequences of SARS-CoV-1 and SARS-CoV-2 S proteins were retrieved from the universal protein knowledgebase (UniProtKB) (Bateman 2019). We performed the pairwise sequence alignment of SARS-CoV-1 (Entry: P59594) and SARS-CoV-2 (Entry: P0DTC2) using the Clustal Omega computer program (https://www.ebi.ac.uk/Tools/msa/clustalo/) and Jalview2 (www.jalview.org). We performed structural alignments of the 3-D structures of SARS-CoV-1 and SARS-CoV-2 using PyMOL (http://www.pymol.org/). The “fetch” and the “align” commands on PyMOL aligned the single chains of the spike proteins of SARS-CoV-1 (PDB ID: 6ACG, chain A) and SARS-CoV-2 (PDB ID: 6VYB, chain A), and RBD of SARS-CoV-1 (PDB ID: 2AJF, chain E) and SARS-CoV-2 (PDB ID: 6M17, chain E).

### Other Computational Prediction Tools

We compared the outputs of six computational prediction tools on SARS-CoV-1 S stability. Each method utilizes different protein structural properties in predicting the effects of mutations on wild-type protein structures. The mutation Cut-off Scanning Matrix (mCSM) tool encodes atomic-distance patterns to predict the impact of mutations on protein structure (Pires et al., 2014a). The Site Directed Mutator (SDM) uses a statistical potential energy function to calculate a stability score. SDM applies a cut-off of 2 kcal/mol to classify stabilizing and destabilizing mutations (Pandurangan et al., 2017). DUET is a tool that combines, consolidates, and optimizes mCSM and SDM tools (Pires et al., 2014b). The overall accuracy of DUET is better than either mCSM or SDM (Pires et al., 2014b). The DynaMut tool utilizes Normal Mode Analysis (NMA) and graph-based signatures to predict the impact of missense mutations (Rodrigues et al., 2018). Finally, the I-mutant suite 3.0 tool uses a support vector machine (SVM) algorithm and accepts the protein sequence or structure as input. However, it predicts the impact of mutations more accurately with the protein structure inputted (Capriotti et al., 2005).

We also compared four computational prediction tools on SARS-CoV-1 S RBD affinity. The mutation Cut-off Scanning Matrix on Protein-Protein Interaction (mCSM-PPI2) tool uses inter-residue network complexes and graph-based signatures to predict the impact of missense mutations on protein affinity (Rodrigues et al., 2019). The Muta-Bind2 tool evaluates the changes caused by missense mutations on protein affinity. Muta-Bind2 is also a useful tool in protein design (Li et al., 2016). Lastly, PISA (Protein, Interfaces, Structures, and Assemblies) tool from the Protein Data Bank in Europe (PDBe) was used to analyze interface residues by comparing the contributions of their solvation energy to the interaction energy (Krissinel and Henrick 2005).

We modified the outputs from these tools to stay consistent with Foldx output. That is, significant positive ΔΔG and ΔΔΔG values destabilize and weaken binding affinity, respectively, and vice versa.

## Results

### Sequence and Structural Alignments of S Proteins of SARS-CoV-1 and SARS-CoV-2

**FIGURE 1:**
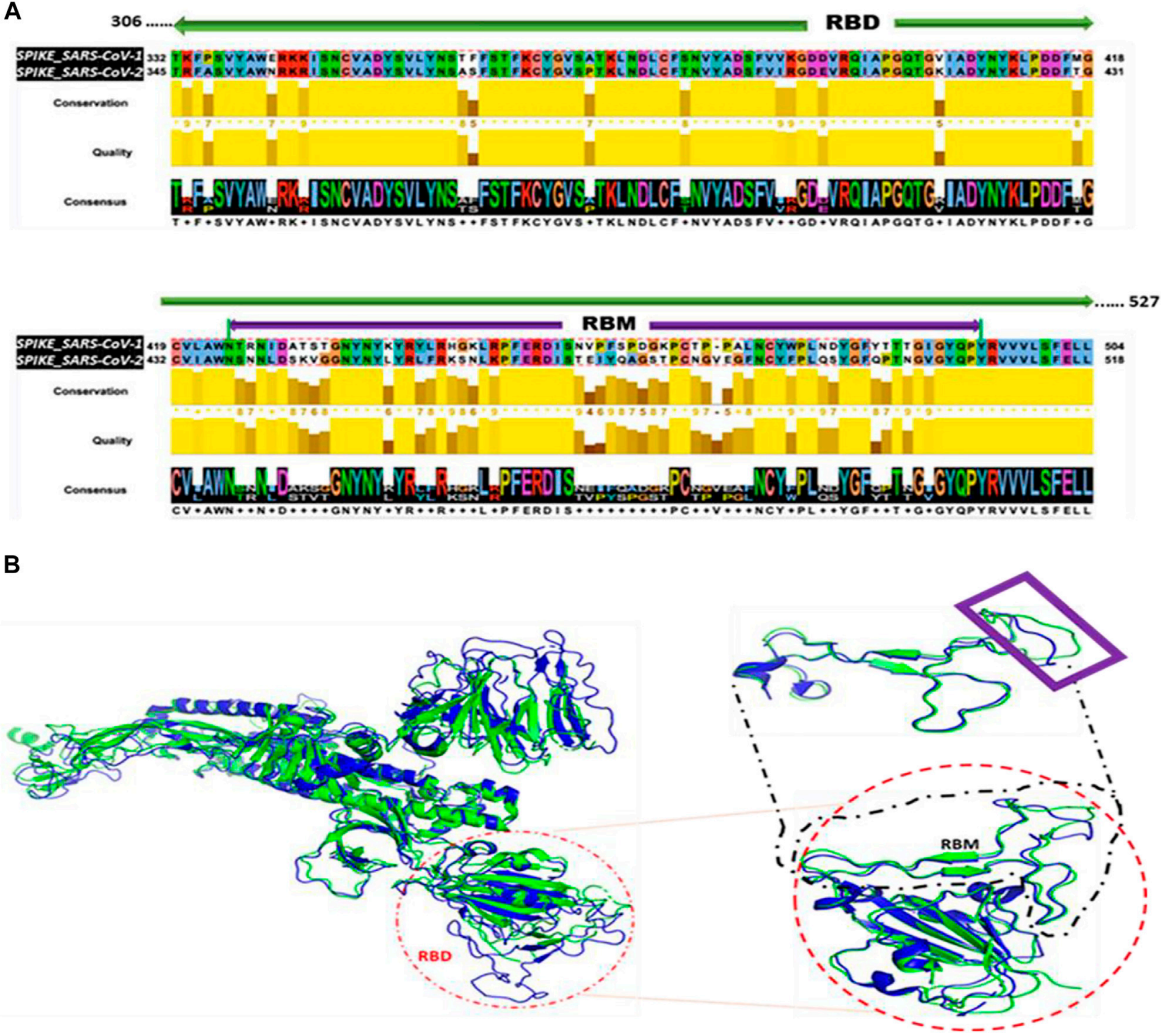
Sequence and Structural Alignment of SARS-CoV-1 S and SARS-CoV-2 S. **(A)** Sequence alignment of the receptor binding domain (RBD) sequences **(B)** Structural alignment. Left: Alignment of the full-length spike proteins of SARS-CoV-1 (Blue) and SARS-CoV-2 (Green). Red circle indicates orientation of RBD. Right: Alignment of RBDs. Black dashed square represents the alignment of the RBMs. Purple rectangle reveals regions with imperfect alignment.

The Jalview tool (Waterhouse et al., 2009) shows the aligned residues, the quality of the alignment, the conservations scores, and the consensus between the RBD sequences of SARS-CoV-1 and SARS-CoV-2 (Figure 1A). The sequence alignment using Clustal Omega algorithm within the Jalview tool indicates a ∼76% sequence identity between RBD regions of SARS-CoV-1 and SARS-CoV-2. The bright yellow bars represent high conservation and quality between the aligned residues within each column. As shown in Figure 1B, the structural alignment revealed the evolutionary relationship between SARS-CoV-1 and SARS-CoV-2. SARS-CoV-1 S (PDB ID: 6ACG, chain A) aligned with SARS-CoV-2 S (PDB ID: 6VYB, chain A) with an RMSD of 2.272. Furthermore, we performed the structural alignment of the RBDs of SARS-CoV-1(PDB ID: 2AJF, chain E) and SARS-CoV-2 (PDB ID: 6M17, chain E), and it yielded an RMSD of 1.043. We selected a shorter fragment, RBM, from the RBD and performed the structural alignment of the RBM of both coronaviruses. This alignment yielded an RMSD of 0.878, which is more homologous. The shape and spatial orientation of the structural alignments overlapped which indicates close atomic coordinates between the two structures.

### Effects of Mutations on Full-Length SARS-CoV-1 S Stability (∆∆G)

**FIGURE 2:**
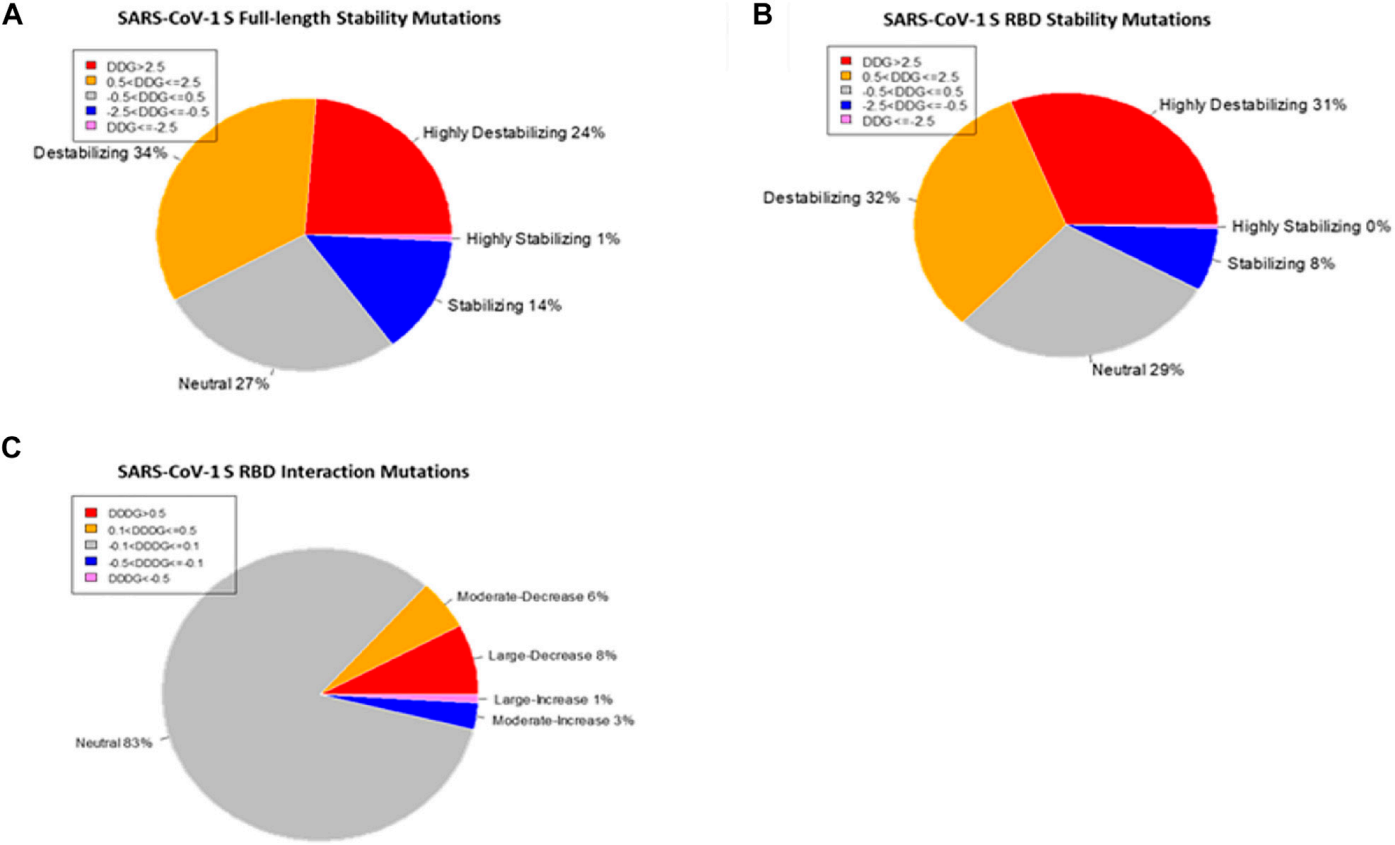
Distribution of the effects of missense mutations on protein stability and binding affinity. Pie charts of the effects of missense mutations on SARS-CoV-1 S (full-length) stability **(A)** and S RBD stability **(B)**. **(C)** Pie chart of the effects of missense mutations on SARS-CoV-1 S RBD binding affinity.

The SARS-CoV-1 S protein has 1,255 residues that were used to generate 23,845 non-redundant missense mutations. The effect of each mutation on the stability of the SARS-CoV-1 S protein was evaluated. Of the total mutations performed, 20,083 missense mutations generated energy changes, while the remaining 3,762 missense mutations gave no output due to missing residues on the protein structure. Figure 2A shows that 11,635 of the 20,083 (58%) missense mutations increased the free energy of the S protein by at least 0.5 kcal/mol, 2,964 of 20,083 (15%) missense mutations reduced the S protein’s free energy by at most –0.5 kcal/mol, and 5,484 of 20,083 (27%) had a neutral effect on the stability of the wildtype S protein. The standard error of energies calculation using the Foldx suite is ∼0.5 kcal/mol (Schymkowitz et al., 2005). Therefore, the folding energy changes within the range (–0.5 < ΔΔG <0.5) are insignificant or categorized as having neutral effect. In more specific categories, 4,767 mutations had a highly destabilizing effect (ΔΔG > 2.5 kcal/mol) on the spike protein, 6,868 mutations moderately destabilize the spike’s protein (0.5 < ΔΔG ≤ 2.5 kcal/mol), 5,484 mutations had a neutral effect (–0.5 < ΔΔG ≤ 0.5 kcal/mol), 2,816 mutations moderately stabilize the S protein (–2.5ΔΔG ≤ –0.5 kcal/mol), and 148 mutations have a highly destabilizing effect (ΔΔG < –2.5 kcal/mol) on the S protein.

**FIGURE 3:**
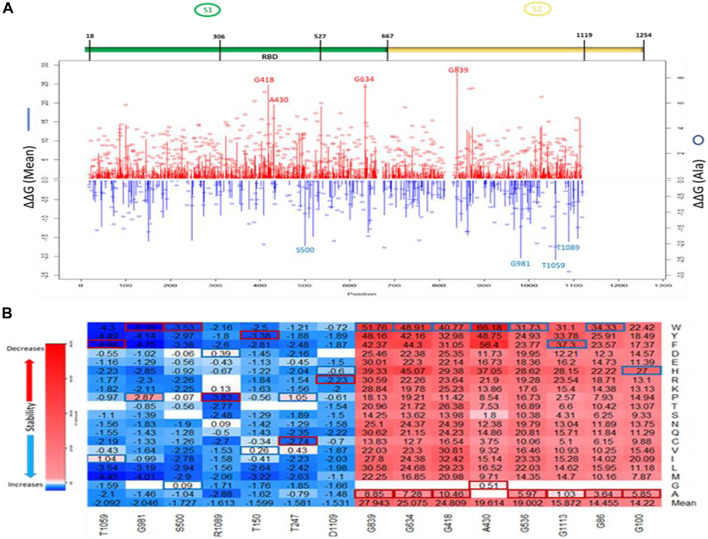
Visualization of ∆∆G values caused by missense mutations on SARS-CoV-1 S (full-length). **(A)** Line chart showing mean ∆∆G values of destabilizing mutations (red upward lines) and stabilizing mutations (Blue downward lines). Red and Blue bubbles show ∆∆G values of Alanine as alternate residues **(Top)**. Overall heatmap of all missense mutations showing different regions/domains **(Bottom)**. **(B)** Heatmap of top five destabilizing mutations and top five stabilizing mutations on SARS-CoV-1 S (full-length). Blue rectangle represents maximum ∆∆G values. Red rectangle represents minimum ∆∆G values.

The line chart and the overall heatmap show the distribution of the mutations along the entire length of the spike protein (Figure 3A). The red lines represent the positive mean values while the blue lines represent the negative mean values. The bubbles represent the folding energy changes when all the residues were mutated to Alanine. Based on the average of ΔΔG, mutations with the highest destabilizing effects were found in positions 839, 634, 418, 430, 536, and 1,113 (Figure 3). The two most destabilizing missense mutations, A430W and A430F, cause energy changes at 66.18 kcal/mol and 56.4 kcal/mol, respectively. This position is also within the RBM of the SARS-CoV-1 spike S protein. On the other hand, mutations with the highest stabilizing effects were found in positions: 1,059, 981, 500, 1,089, 150, and 247 (Figure 3). The two most stabilizing missense mutations, G981W and T1059F, reduced the free energy of the wildtype structure by –5.16 kcal/mol and –4.98 kcal/mol, respectively. The residue positions with the highest and lowest mean folding energy changes (∆∆G) were G839 and T1059, which are both within the S2 subunit of the S protein. Compared to the mean values of all mutations, the missense mutations to Alanine had more destabilizing effects than stabilizing effects. The two “white” gaps in the overall heatmap represent missing values in the residue positions 661–673 and 812–831, respectively.

### Effects of Mutations on SARS-CoV-1 S RBD Stability (∆∆G)

**FIGURE 4:**
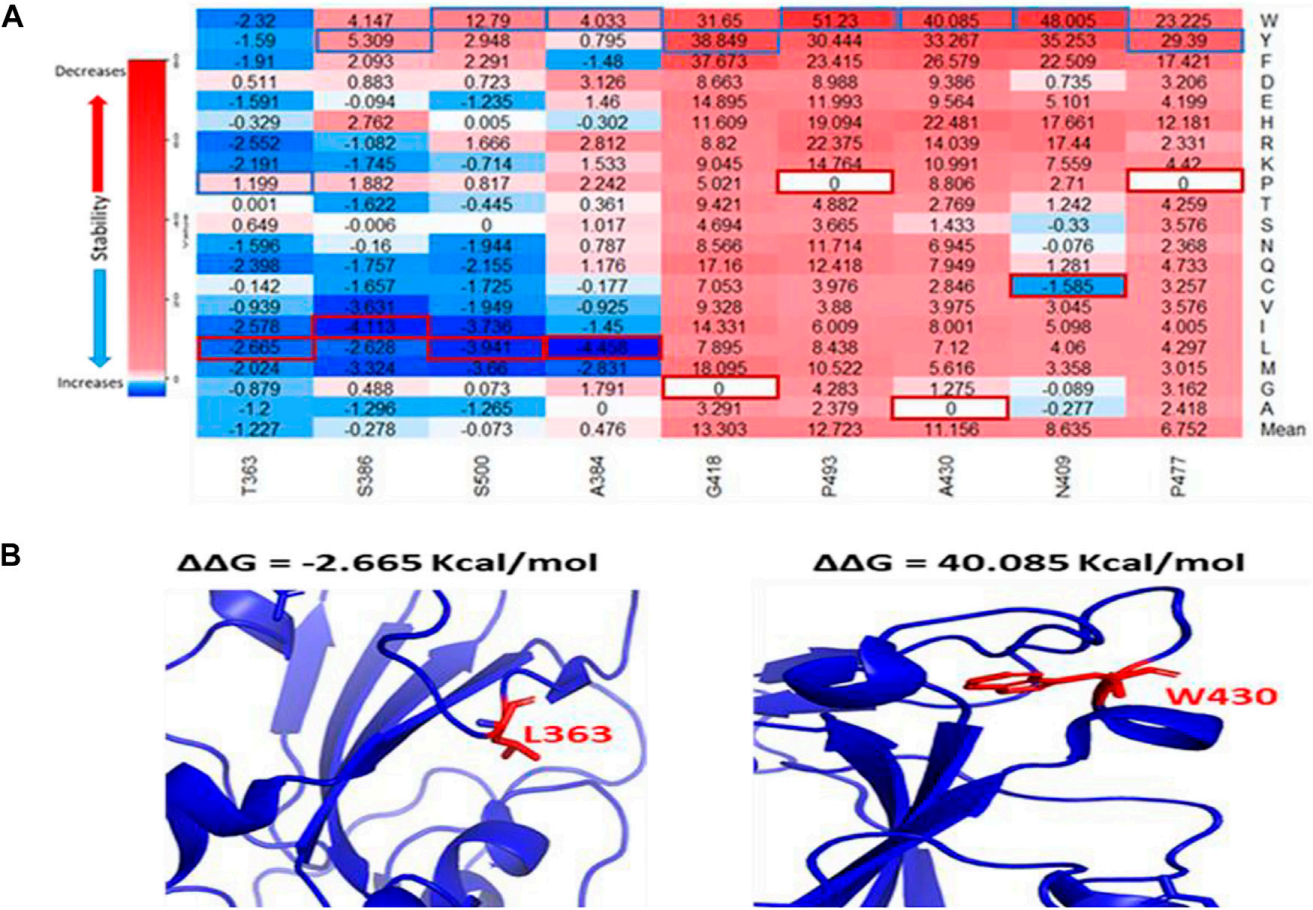
Effects of the target missense mutations on SARS-CoV-1 S RBD protein stability. **(A)** Heatmap of top destabilizing mutations and top stabilizing mutations (∆∆G) Values up the scale (red) decreases stability/binding affinity and vice versa (blue). The blue rectangular box represents maximum values. The red rectangular box represents minimum values. **(B)** Structural representation of SARS-CoV-1 S RBD showing stabilizing (T363L) and destabilizing (A430W) mutations in red and their side chains.

We utilized the crystal structure of the RBD of SARS-CoV-1 (PDB ID:2AJF) for computational prediction of the effect of computed mutations on the stability of SARS-CoV-1 RBD. This analysis gave an alternative perspective on the predictive power of computational tools. We generated 3,841 mutations and calculated the folding energy change (∆∆G) caused by each missense mutation. Like the full spike analysis, 63% of the missense mutations destabilized the SARS-CoV-1 RBD structure. Meanwhile, 8% of the missense mutations stabilized the SARS-CoV-1 RBD structure. Figure 2B shows the pie chart of the effects of the missense mutations on stability of SARS-CoV-1 S RBD. The distribution of the effect of all missense mutations in both the RBD and the full-length S stability analysis correlates (r = 0.6824). Observation of individual mutations revealed analytic similarity in both stability analysis. The heatmaps in Figures 3B, 4A also show similarity in mis-sense mutations with large effects on the stability of the SARS-CoV-1 S stability. For example, the missense mutation A430W had a large effect on both the stability of the RBD (Figure 4B) and the entire spike protein of the SARS-CoV-1.

### Comparison of Effects of Mutations on Stabilities of Full-Length S Proteins of SARS-CoV-1 and SARS-CoV-2

We compared the mutational effects of seven residues on SARS-CoV-1 S protein to corresponding residues on SARS-CoV-2 S protein. Four residues (T1059, G981, S500, and R1089) had the highest mean stabilizing effects, while three residues (G839, G634, and A430) had the highest mean destabilizing effects. Pairwise alignment of SARS-CoV-1 S and SARS-CoV-2 S showed that the residues T1059, G981, S500, R1089, G839, G634, and A430 on SARS-CoV-1 corresponds with residues T1077, G999, S515, R1107, G857, G648, and S443 on SARS-CoV-2, respectively. As shown in Table 1, the missense mutations on SARS-CoV-1 S protein had similar effects on its stability when compared to corresponding residues on SARS-CoV-2 S protein. We observed that the missense mutation A430S destabilizes SARS-CoV-1 S protein by introducing the folding energy change at 1.8 kcal/mol. Consistently, S443A in corresponding position of SARS-CoV-2 increase the S stability (∆∆G = –0.768 kcal/mol). Interestingly, SARS-CoV-1 D600G can destabilize S (∆∆G = 0.21 kcal/mol). D614G, the dominant variant of SARS-CoV-2, corresponds to D600G but was predicted to stabilize S (∆∆G = –0.784 kcal/mol). Another In position 247, the substitution of Threonine with Alanine increased the stability of the SARS-CoV-1. However, the corresponding residue on SARS-CoV-2 A260 was missing. Furthermore, we compared the predicted stability effects of target SARS-CoV-2 S RBD mutations with the current deep mutational scanning approach (Starr et al., 2020). The computational predictions agree with the experimental results (Table 1).

**TABLE 1:** Mapping and comparing the effects of SARS-CoV-1 and SARS-CoV-2 missense mutations on protein stability (Top) and binding affinity (Bottom).

SARS-CoV-1			SARS-CoV-2			
**Protein stability**						
**MUTATION**	**∆∆G (kcal/mol)**	**Effect**	**MUTATION**	**∆∆G (kcal/mol)**	**Effect**	**DMS ∆∆G Effect**
G839W	51.76	Decrease	G857W	58.212	Decrease	NA
G839Y	48.16	Decrease	G857Y	43.523	Decrease	NA
G634W	48.91	Decrease	G648W	43.326	Decrease	NA
A430W	66.18	Decrease	S443W	33.41	Decrease	Decrease
A430F	56.4	Decrease	S443F	22.173	Decrease	Decrease
A430S	1.8	Decrease	S443A	–0.768	Increase	Increase
A430Y	48.75	Decrease	S443Y	25.052	Decrease	Decrease
D600G	0.21	Decrease	D614G	–0.784	Increase	NA
T487Y	6.09	Decrease	N501Y	–1.038	Increase	Increase
T1059F	–4.98	Increase	T1077F	3.263	Decrease	NA
G981W	–5.16	Increase	G999W	27.099	Decrease	NA
G981F	–4.75	Increase	G999F	25.374	Decrease	NA
S500W	–3.53	Increase	S514W	–3.303	Increase	Increase
T247C	–2.74	Increase	A260C	NA	NA	NA
T247A	–0.79	Increase	A260T	NA	NA	NA
**Binding Affinity**						
**MUTATION**	**∆∆∆G (kcal/mol)**	**Effect**	**MUTATION**	**∆∆∆G (kcal/mol)**	**Effect**	**DMS ∆∆∆G Effect**
G488P	15.921	Decrease	G502P	11.767	Decrease	Decrease
T487Y	20.018	Decrease	N501Y	4.55	Decrease	Increase
T487N	2.284	Decrease	N501T	–1.346	Increase	Increase
T487W	13.42	Decrease	N501W	3.118	Decrease	Increase
G482E	5.758	Decrease	G496E	7.278	Decrease	Decrease
S432Y	–1.686	Increase	V445Y	–0.253	Increase	Neutral
S432V	–0.22	Increase	V445S	–0.0045	Neutral	Neutral
N479M	–1.42	Increase	Q493M	–0.3	Increase	Increase
N479Q	–0.607	Increase	Q493N	0.679	Decrease	Decrease
P462D	–0.97	Increase	A475D	0.633	Decrease	Decrease
P462A	0.391	Decrease	A475P	0.282	Decrease	Decrease

*Note:* Result from Deep Mutational Scanning (DMS) approach was included to compare with the effects on SARS-CoV-2 S RBD, from Foldx.

### Effects of Mutations on SARS-CoV-1 S RBD Binding Affinity (∆∆∆G)

**FIGURE 5:**
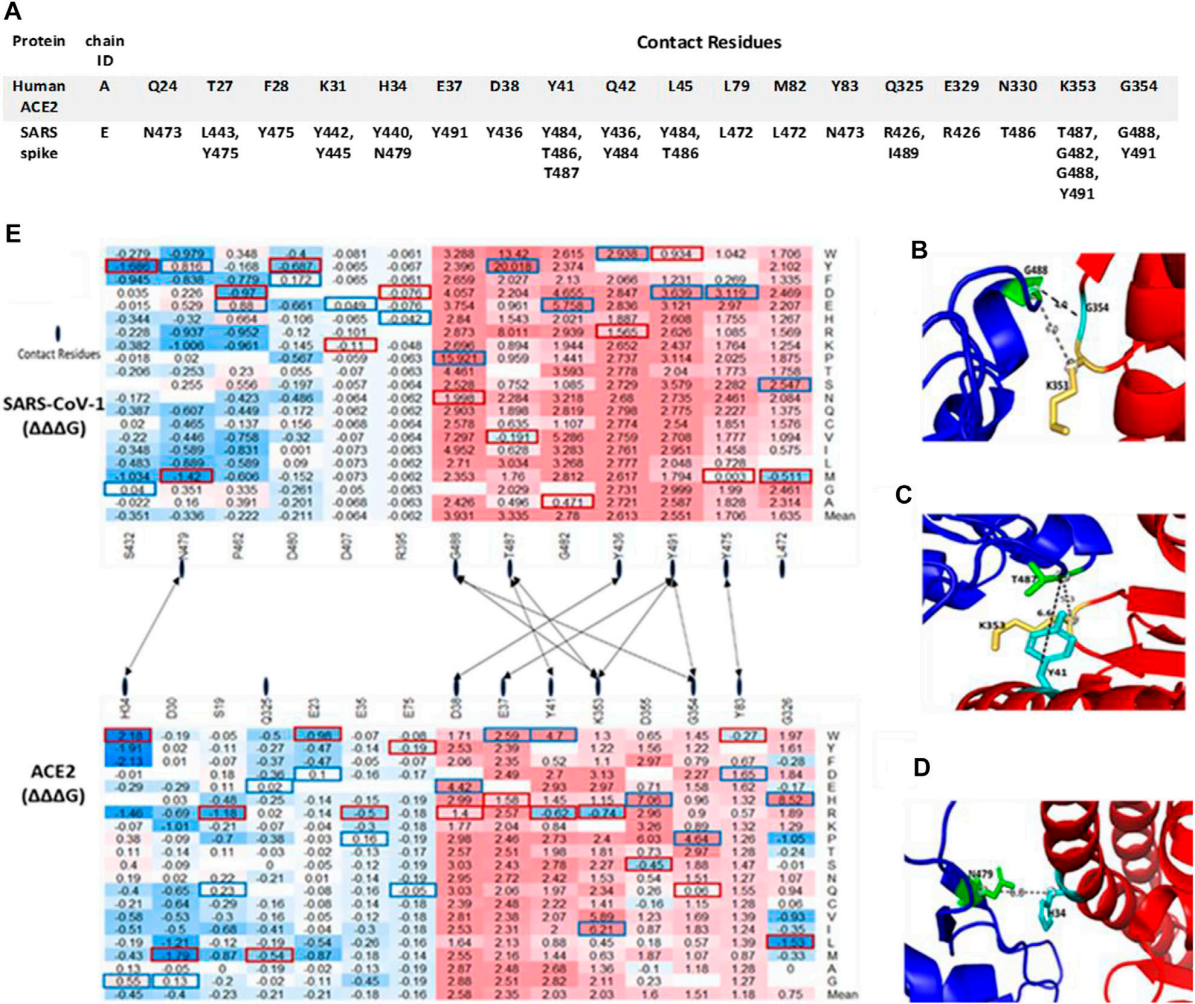
Top missense mutations on SARS-CoV-1 S RBD-ACE2 interface. **(A)** Contact residues between Human ACE2 **(Top)** and SARS-CoV-1 S RBD protein **(Bottom)**. **(B–D)** Structural representation of important binding residues (SARS-CoV-1 S RBD interacting residue in green and hACE2 interacting residues in yellow and cyan). **(B)** RBD G488 interacts with K353 and G354 on ACE2. **(C)** RBD T487 interacts with Y41 and K353 on ACE2. **(D)** RBD N479 interacts with H34 on ACE2. Dotted lines indicate distance between two residues. **(E)** Heatmap of the ∆∆∆G of target S RBD mutations. Dotted lines indicate contact residues between the SARS-CoV-1 S RBD and hACE2.

The protein structure (PDB ID:2AJF) used for interaction analysis covered the S RBD that interact with the hACE2 receptor in humans. This RBD chain contains 180 residues, which spanned from residual position 323 to 502. A total of 3,420 mutations were computed, with 114 missing values. The remaining 3,306 mutations were classified into one of five categories according to their binding energy changes (ΔΔΔG). As shown in Figure 2C, 257 mutations had a large-decrease effect, 183 mutations had a moderate-decrease effect, 2,738 mutations had a neutral effect, 97 mutations had a moderate-increase effect, and 31 mutations had a large-increase effect. Figure 5E shows the mutations with the largest effects, based on ΔΔΔG mean values from all possible mutations in that position.

**FIGURE 6:**
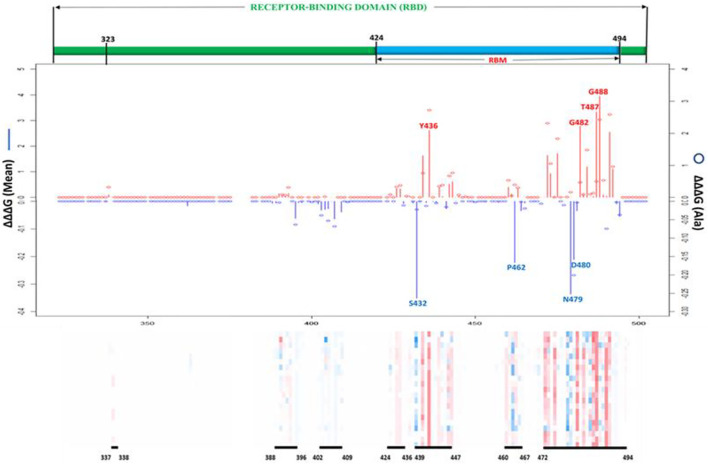
Effects of all possible missense mutations on SARS-CoV-1 S RBD binding affinity. Line chart showing mean ∆∆∆G values of mutations decreasing binding affinity (red upward lines) and mutations increasing binding affinity (Blue downward lines). Red and Blue bubbles show ∆∆G values of Alanine as alternate residues **(Top)**. Overall heatmaps showing seven hot regions/domains and residue span **(Below)**.

The seven topmost mutations with decreasing effects are found in positions which have mean values greater than 1.5 kcal/mol. While the six topmost mutations with in-creasing effects are found in positions which have mean values less than –0.3 kcal/mol. The values in red boxes represent the minimum values in each position, while the values in blue boxes represent maximum values. The overall distribution of the missense mutations as they change ACE2 binding affinity of the SARS-CoV-1 S protein can be seen in Figure 6. There are 7 distinct regions or domains in the heatmap: [337–338], [388–396], [402–409], [424–436], [439–447], [460–467], and [472–494]. Four of the seven distinct regions are concentrated within the RBM, and they span longer stretches of residues. Most of the red and blue spikes on the line chart are also within the RBM. These distinct regions can provide insight into which residues play a key role in the interaction between SARS-CoV-1 S protein and the hACE2 receptors in humans.

### Comparison of Effects of Mutations on the Binding Affinities of SARS-CoV-1 and SARS-CoV-2 S Protein

As shown in Table 1, Asparagine is found on position 501 in SARS-CoV-2 which corresponds to Threonine on position 487 in SARS-CoV-1. The computed missense mutation T487N increased the ∆∆∆G by 2.28 kcal/mol and decreased the ∆∆G by –0.14. In contrast, N501T, on the SARS-CoV-2 decreased ∆∆∆G by –1.346 kcal/mol. Tyrosine and Tryptophan have nonpolar aromatic chains therefore the missense mutations T487Y and T487W, had similar effects (decrease binding affinity) on corresponding residue on SARS-CoV-2, N501Y and N501W, respectively. Most of the missense mutations on positions S432, P462, and N479, on the average, increase the binding affinity of SARS-CoV-1. However, the corresponding residues on SARS-CoV-2 are Valine, Alanine, and Glutamine, respectively. The effect of missense mutations on these residues were evaluated by examining missense mutations on corresponding positions on SARS-CoV-1. However, mis-sense mutation N479Q decreased the ∆∆∆G by –0.607 kcal/mol and increased ∆∆G by 1.91 kcal/mol. A possible explanation is that the missense mutations created Van der Waal clashes with neighboring atoms because of shorter distance; The missense mutation, T487Y, caused a differential Van der Waals of -0.449 kcal/mol, which led to a greater re-pulsion from nearby atoms. Recent SARS-CoV-2 variant L452R (∆∆∆G = –0.395 kcal/mol; ∆∆G = 0.021 kcal/mol) corresponds to SARS-CoV-1 K439R (∆∆∆G = 0.247 kcal/mol; ∆∆G = 0.41 kcal/mol). The change in residue from Lysine in SARS-CoV-1 S protein to Leucine in SARS-CoV-2 S protein may be responsible for the increase in binding affinity caused by L452R.

### Effects of Mutations on Post-translational Modification Sites of S Protein

Post-Translational modifications (PTMs) of the SARS-CoV-1 S protein are responsible for the folding, maturation, and function of the S protein. An important PTM utilized by SARS-CoV-1 is the O- and N-linked Glycosylation, which plays a key role in the shielding of viruses from the host’s immune system (Watanabe et al., 2019). As a result, viruses evolve to become glycosylated as much as possible (Sugrue 2007). One O-linked glycosylation site, S336, located within the RBD was predicted using online webserver (Steentoft et al., 2013). On the average, the computed missense mutations in this position increase the stability of SARS-CoV-1. For example, the missense mutation, S336E, reduces the ∆∆G by -1.93 kcal/mol, thereby stabilizing the SARS-CoV-1 spike protein. Conversely, S336V in-creases the ∆∆G by 1.28 kcal/mol, thereby destabilizing the SARS-CoV-1 structure. We used five online prediction tools in our N-linked glycosylation sites prediction- NetNGly 1.0 (Kumar et al., 2020), N-GlyDE (Pitti et al., 2019), SPRINT-Gly (Taherzadeh et al., 2019), Glycopp v1.0 (Chauhan et al., 2012), and Glycopred (Hamby and Hirst 2008). We narrowed our results to sites predicted by at least two or three prediction tools. Of these, we selected 20 N-linked glycosylation sites located within the spike protein of the SARS-CoV-1. Three putative N-linked glycosylation sites, N318, N330, N357, were located within the RBD, and they were predicted to have no effect on the binding affinity of SARS-CoV-1 spike protein to hACE2. On the average, mutations at position N318 were predicted to increase the stability of SARS-CoV-1. However, on the average, the mutations on N330 and N357 were predicted to have neutral effects.

**FIGURE 7:**
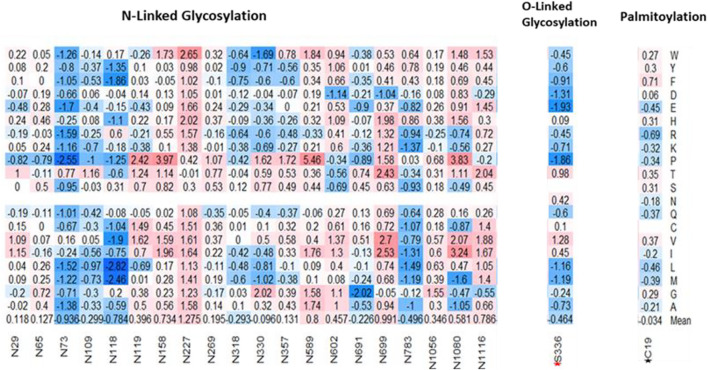
Effects of missense mutations on putative post translational sites on SARS-CoV-1 full-length S protein. **(Left-Right)** 20 N-Glycosylation sites, 1 O-Glycosylation site (S336), and 1 Palmitoylation site (C19).

Figure 7 shows the target mutations in twenty N-linked glycosylation sites, one O-linked glycosylation site, and one palmitoylation site. Palmitoylation modifies the spike protein through cysteine-rich residues (Petit et al., 2007). This is known to mediate the fusion of the spike protein to hACE2. Predicted palmitoylation sites (Ren et al., 2008) were found in positions C19, C1217, C1218, C1222, C1232, C1235, and C1236. The average computed mutations at the C19 position predicted neutral effects, which showed no significant effect in the stability of SARS-CoV-1. However, the remaining six palmitoylated were located outside the residues covered by the structural protein.

### Statistical Analysis of SARS-CoV-1 S Protein Mutation Pathogenicity

**FIGURE 8:**
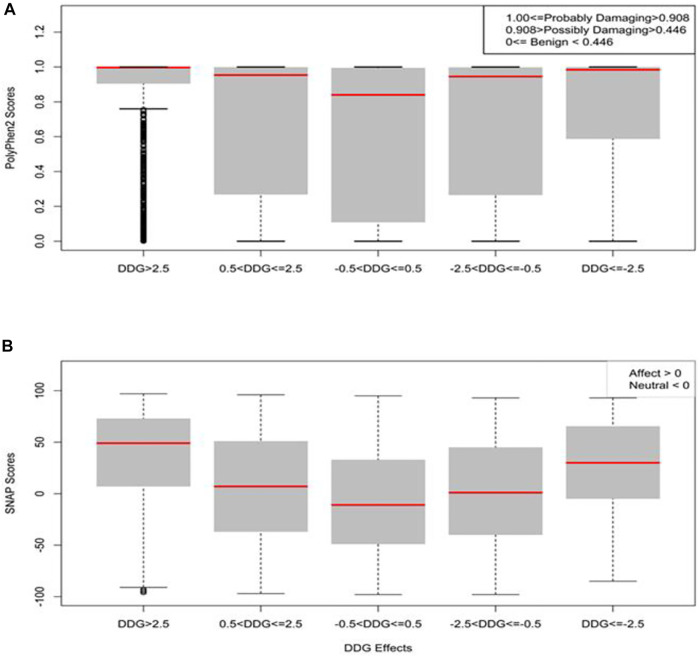
Boxplots of the prediction of mutation pathogenicity on full-length Spike. **(A)** PolyPhen2 scores and **(B)** SNAP scores against five categories of the effects of mutations on SARS-CoV-1 full-length S protein stability.

We used sequence-based mutation pathogenicity tools to predict the damaging effect of our computed mutations on the SARS-CoV-1 S function. We analyzed 25,101 missense mutations using the full-length SARS-CoV-1 S (1–1,255). The PolyPhen2 scores gave probabilistic values on the tolerance and deleterious effect of missense mutations. A score less than 0.446 is considered benign, a score greater than 0.446 but less than 0.908 is considered possibly damaging, and a score greater than 0.908 is considered probably damaging. In Figure 8A, the missense mutations with neutral effect were predicted to be mostly tolerated with some classified as benign, while the mean value, as shown by the red line, is considered possibly damaging. Whereas the mean values of the moderately increasing and decreasing mutations were predicted to be possibly damaging. However, the mean values of large increasing and decreasing mutations were predicted to be probably damaging. The analysis of variance (ANOVA) showed that the means of all five categories were significantly different, with *p*-value < 2e-16 (Figure 8A).

SNAP is a neural network machine learning algorithm that accepts protein structure as input for functional predictions (Bromberg and Rost 2007). SNAP scores less than zero have neutral effect, while SNAP scores greater than zero have a pathogenic effect. 13,179 (∼53%) of the total 25,101missense mutations have pathogenic effects. We performed further analysis to correlate the effects of folding energy change ∆∆G on stability with their SNAP scores. As shown in Figure 8B, the SNAP scores of missense mutations with effects greater than 2.5 kcal/mol or less than –2.5 kcal/mol were higher than missense mutations with moderate effects (0.5 < ΔΔG ≤ 2.5 or –2.5 =< ΔΔG < –0.5 kcal/mol). Furthermore, the missense mutations with neutral effects on SARS-CoV-1 stability also gave a neutral SNAP prediction. The statistical analysis showed that the correlation of SNAP scores in the five groups were significant with a *p*-value of <2e-16.

### Computational Analysis of S Viral Mutations in SARS-CoV-1 Isolates

This study analyzed mutations, found on the SARS-CoV-1 protein, which have been verified through experiments to have cellular or molecular effects on its functions. Analysis of SARS-CoV-1 isolates from the 2002–2003 and 2003–2004 outbreaks revealed the main driving mutations (Li W. et al., 2005). Six residue positions of S protein, 344, 360, 472, 479, 480, and 487, were highlighted to have varying residues between the two isolates (Li W. et al., 2005). The 2002–2003 isolate had residues K344, F360, L472, N479, D480, and T487. Meanwhile, the 2003–2004 isolate had the following corresponding residues R344, S360, P472, K479, G480, and S487. The effects of the residue changes from the 2002–2003 isolate to the 2003–2004 isolate are shown in Table 2. The residue change, N479K, would increase the binding affinity (∆∆∆G = –1.008 kcal/mol) and reduced S stability (∆∆G = 1.52 kcal/mol). Two mutations, L472P and T487S, decreased the binding affinity of S RBD-ACE2 by introducing ∆∆∆G at 1.875 kcal/mol and 0.752 kcal/mol, respectively. Furthermore, the mutation L472P destabilizes SARS-CoV-1 (∆∆G = 1 kcal/mol). Therefore, understanding how the changes in these residues affect the stability and infectivity of the SARS-CoV-1 in the two isolates would help in the target of specific residues on SARS-CoV-2. Recent research to study the mutation of residues from the 2002–2003 isolates to residues in 2003–2004 isolate is consistent with this computational study (Li W. et al., 2005).

**TABLE 2:** Computational prediction of the effect of residue changes from 2002 to 2003 isolate to 2003–2004 isolate. (N) = Neutral, (D) = decrease, (I) = increase.

SARS-CoV-1		Energy change			PolyPhen2		SNAP	
2002–2003 isolate	2003–2004 isolate	Mutation	∆∆∆G (kcal/mol)	∆∆G (kcal/mol)	Score	Prediction	Score	Effect
K344	R344	K344R	0 (N)	–0.02 (N)	0	Benign	–89	Neutral
F360	S360	F360S	0 (N)	1.5 (D)	0	Benign	–77	Neutral
L472	P472	L472P	1.875 (D)	1 (D)	0.432	Benign	54	Pathogenic
N479	N479	N479K	–1.008 (I)	1.52 (D)	0.598	Possibly damaging	–80	Neutral
D480	G480	D480G	–0.261 (I)	–0.25 (N)	0.001	Benign	–27	Neutral
T487	S487	T487S	0.752 (D)	–0.35 (N)	0.003	Benign	–89	Neutral

### Comparison of Different Computational Prediction Tools on Target Mutations

#### Protein Stability

Of the four target destabilizing mutations predicted by Foldx, four other computational tools predicted G634W as a destabilizing mutation, while three other tools except, DynaMut and I-mutant3, predicted A430W, A430Y, and G839W as destabilizing mutations. Of the two target stabilizing mutations predicted by Foldx, only two other tools (DynaMut and SDM) predicted G981W and T1059F as stabilizing mutations. Two of the three key mutations (T487S and L472P) on 2002–2003 viral isolates were predicted to de-stabilize the protein S protein by four of the six prediction tools. However, N479K predict-ed as a destabilizing mutation by Foldx, DynaMut, and I-mutant3. Meanwhile, DUET, mCSM, and SDM predicted N479K as a stabilizing mutation (Table 3).

**TABLE 3:** Comparison of the prediction of target mutations among different computational tools on SARS-CoV-1 S stability. (N) = Neutral, (D) = Destabilize, (S) = Stabilize.

Stability ∆∆G (kcal/mol)												
**Mutations**	**Foldx**	**Effect**	**DUET**	**Effect**	**mCSM**	**Effect**	**DynaMut**	**Effect**	**SDM**	**Effect**	**I-mutant3**	**Effect**
A430W	66.180	D	1.675	D	1.451	D	–0.729	S	1.76	D	0.41	N
A430Y	48.750	D	1.465	D	1.055	D	–1.588	S	1.88	D	0.47	N
G839W	51.760	D	1.949	D	1.633	D	–0.989	S	2.11	D	0.4	N
G634W	48.910	D	2.02	D	1.55	D	0.271	D	2.31	D	0.5	N
G981W	–5.160	S	0.759	D	1.327	D	–2.179	S	–1.09	S	0.63	D
T1059F	–4.980	S	0.827	D	1.008	D	–0.729	S	–0.34	S	0.73	D
^a^T487S	–0.350	N	0.809	D	0.861	D	1.067	D	0.98	D	0.39	N
^a^N479K	1.520	D	–0.759	S	-0.148	S	0.312	D	–0.64	S	0.97	D
^a^L472P	1.000	D	0.114	D	0.345	D	0.106	D	–0.05	S	0.37	N

a Key mutations from 2002 to 2003 viral isolates.

#### Protein Affinity

As shown in Table 4, T487S and L472P were predicted to decrease or weaken SARS-CoV-1 S RBD affinity to ACE2 by Foldx, mCSM-PPI2, and MutaBind2. However, N479K was predicted by mCSM-PPI2 and MutaBind2 to decrease the binding affinity of SARS-CoV-1 S RBD. Despite predicting that all three mutations decrease the binding affinity of SARS-CoV-1 S RBD, MutaBind2 predicted that the three mutations were not deleterious. We used PDBePISA to evaluate the solvation energy change caused by a mutation and found the mutation effects are consistent with Foldx predictions. Of the three mutations, only N479K increases the solvation energy by 0.29 kcal/mol, which indicate this mutation can increase the interaction force or binding affinity of the residue.

**TABLE 4:** Comparison of the prediction of target mutations among different computational tools on SARS-CoV-1 S RBD binding affinity.

	Binding affinity ∆∆∆G (Kcal/mol)						
	Foldx		mCSM-PPI2		MutaBind2		PDBePISA	
Mutations	∆∆∆G	Effect	∆∆∆G	Effect	∆∆∆G	Effect	∆∆^i^G	Effect
T487S	0.752	Decrease	0.888	Decrease	1.28	Decrease	–0.37	Decrease
N479K	–1.009	Increase	1.448	Decrease	0.76	Decrease	0.29	Increase
L472P	1.875	Decrease	0.49	Decrease	0.03	Decrease	–1.00	Decrease

*Note*: ∆∆^i^G is the change in solvation energy.

## Discussion

Coronaviruses have been the cause of the most recent pandemics. The most recent coronaviruses are SARS-CoV-1 and SARS-Cov-2. A recent study showed a close relationship between the sequence and the structure of SARS-CoV-1 and SARS-CoV-2 (Kumar et al., 2020). The structural alignment performed in our study also revealed an evolutionary relationship between their S proteins, RBDs, and RBMs. However, the orientation of SARS-CoV-1 residues, S461 – N473, did not align properly with SARS-CoV-2 residues, A475 – N487. This imperfect alignment could be responsible for their varying binding affinities to the human hACE2 receptor (Oostra, de Haan, and Rottier 2007).

The stability of the S protein is crucial for the rapid transmissions of infection (Moreira et al., 2020). Understanding the role of mutations on S protein stability would help in designing therapeutic drugs and vaccines. Our prediction showed that more than half of the SARS-CoV-1 S mutations (∼68%) destabilize the full-length S protein. Most of these destabilizing mutations involved the substitution of Glycine and Alanine residues, which are amino acids with hydrophobic side chains, with residues with longer hydro-phobic side chains. Glycine has a short side chain which may hinder interactions with neighboring residues. Alanine has a deleted side chain which also makes it difficult to interact with neighboring residues. However, Alanine and Glycine exhibit hydrophobic effects which help to stabilize protein structures. The two most destabilizing missense mutations are A430W and A430F. The long side chains of Phenylalanine and Tryptophan disrupt the hydrophobic core of the SARS-CoV-1 S protein structure by introducing steric clashes. While most highly stabilizing mutations involved amino acids with polar side chains, such as Threonine, Arginine, and Serine, except for Glycine. The two most stabilizing missense mutations are G981W and T1059F (Figure 3B). Their polar side chains al-low hydrogen bonding with water and ionic bond with nearby polar molecules. The pre-diction of the effects of mutations on the stability of SARS-CoV-1 S RBD revealed similar results with the analysis on full-length S protein. Among the top five positions with the highest average destabilizing effect, G418 and A430 were common (Figure 4A). Our study reveals a high correlation in the effects of mutations on the S RBD and the full-length S protein. This correlation shows that a mutation introduced into the SARS-CoV-1 will have a similar impact regardless of the parts, RBD or full-length S protein, used by the SARS-CoV-1. Due to the high similarity between SARS-CoV-1 and SARS-CoV-2, we compared the effects of mutations on their S proteins stabilities. After extrapolating the top results, we found out that the effects of mutations on protein stability are similar in corresponding positions on SARS-CoV-1 and SARS-CoV-2. We also looked at the residues that are different in corresponding positions by substituting them with each other. For instance, A430 on SARS-CoV-1 corresponds to S443 on SARS-CoV-2. The mutation A430S destabilizes SARS-CoV-1 S protein, while the mutation S443A stabilizes SARS-CoV-2 S protein. Interestingly, T487N decrease the binding affinity of SARS-CoV-1, while N501T increase the binding affinity of SARS-CoV-2 (Table 1). A previous study performed deep mutation scanning on SARS-CoV-2 identified N501T as a binding affinity enhancer (Starr et al., 2020). Overall, our findings agree with the results from deep mutational scanning analysis. D614G and N501Y are mutations found in the Delta/B.1.617.2 variant of SARS-CoV-2 S protein. Interestingly, D600G and T487Y destabilize SARS-CoV-1 S protein, while corresponding D614G and N501Y stabilize SARS-CoV-2 S protein. However, the Foldx predicted T487Y to weaken the binding affinity between the SARS-CoV-S RBD and hACE2.

The binding of the S protein to the ACE2 allows SARS-CoV-1 to enter the host’s cells (Wan et al., 2020). In this study, we predicted the effects of S mutations on the binding affinity between SARS-CoV-1 S RBD and hACE2. The top missense mutations decreasing binding affinity are T487Y, T487W, and G488P, with binding-energy changes (ΔΔΔG) of 20.018 kcal/mol, 13.42 kcal/mol, and 15.921 kcal/mol, respectively. These mutations occur on neighboring residues in the RBM of the SARS-CoV-1 S protein. By contrast, S432Y and N479M have the minimum binding-energy changes (ΔΔΔG) of –1.686 kcal/mol and –1.42 kcal/mol, respectively. Both mutations can strengthen the binding of SARS-CoV-1 S to hACE2. Furthermore, we looked at the Van der Waals interaction distance between inter-acting residues on SARS-CoV-1 S RBD and hACE2. The distance between residue T487 and K353 decreased when Threonine mutates to Tyrosine. On residue G488, all possible mutations will weaken its binding affinity for residues K353 and G354 on the hACE2 receptor. However, most missense mutations on SARS-CoV-1 N479 will strengthen its binding for H34 on hACE2 (Figures 5B–D). Compared to SARS-CoV-2 S RBD, the effects of mutations on the binding affinity of SARS-CoV-1 S RBD are similar. A few exceptions are in regions where the corresponding residues differ. For example, missense mutation S432V on SARS-CoV-1 decreased the binding energy by –0.22 kcal/mol. However, S432V has a neutral effect on its stability. Meanwhile, on SARS-CoV-2 S RBD, the missense mutation V445S does not affect its binding affinity.

SARS-CoV-1 modifies its S protein through N-linked Glycosylation, O-linked glycosylation, and palmitoylation. The S protein of SARS-CoV-1 possesses glycosylation sites like other coronaviruses (Kumar et al., 2020). These modifications allow SARS-CoV-1 to bind differentially to hACE2 receptor, and to evade the immune system. Analysis of the effect of mutations on 20 N-Glycosylation sites and one palmitoylation site shows that these sites are crucial for the function of SARS-CoV-1. Unlike the O-linked glycosylation site, S336, our results showed that most mutations in the N-linked glycosylation sites would destabilize the SARS-CoV-1 S protein.

Furthermore, we used Polyphen2 and SNAP scores to predict the pathogenicity effects of the mutations, respectively. These tools have proven to identify non-synonymous substitutions with a high accuracy. In a study, Polyphen-2 achieved a prediction rate of 92% (I. Adzhubei, Jordan, and Sunyaev 2013). In a different study, SNAP identified all neutral and non-neutral substitutions with an 80% accuracy (Bromberg and Rost 2007). In this study, all five categories of the effects of mutations were significantly different, with *p*-value<2e-16. The Polyphen2 and SNAP scores of mutations with neutral effect were lower compared to the other four categories. The outcome of PolyPhen2 and SNAP predictions indicate the reliability of folding energy change (ΔΔG) in predicting the effect of missense mutations on the stability of SARS-CoV-1 S protein.

With our computational result, we compared viral isolates from 2002 to 2003 and 2003–2004 outbreaks. A previous study suggested that changes in residues affect the affinity of SAR-CoV-1 for hACE2 (Li W. et al., 2005). In comparison, the decreasing order of affinity to hACE2 is 2002–2003 SARS-CoV-1 isolate > SARS-CoV-2 > 2003–2004 SARS-CoV-1 isolate (Wan et al., 2020). Therefore, we were curious to know the residue change within the SARS-CoV-1 RBD that resulted in a less severe 2003–2004 viral isolate. Our computational mutagenesis on the six residues in a previous study highlighted their role in hACE2 binding. The residues P472 and S487 weaken the binding affinity of the 2003–2004 viral isolate. The substitution of L472 with P472 in the 2003–2004 viral isolate reduces the binding of SARS-CoV-1 to L79 and M82 residues on hACE2. Threonine at position 487 has a stronger affinity to residues Y41, K353, and D355 on hACE2 than Serine at position 487. This is due to the methyl group in Threonine (Li W. et al., 2005). Also, the residues S360, P472, and K479 destabilize the 2003–2004 viral isolate. With the use of several prediction tools, the key mutations in the 2002–2003 viral isolates did not result in big changes in ΔΔG and ΔΔΔG. However, the simultaneous substitutions of these changes in residues might have contributed to a less severe 2003–2004 viral isolate. Also, the biological validation for effects of these viral mutations on protein stabilities and virus-receptor interactions are required. Further, we compared key mutations derived from Foldx with other reliable computational tools, and the predictions were highly correlated. Foldx uses a force field to create a rotamer database which considers different rotations and conformations of the protein. This feature makes Foldx a reliable computational tool.

Other structural proteins such as nucleocapsid (N), membrane (M), and envelope (E) proteins play crucial roles in the function of coronaviruses. Saturated computational mutagenesis can be used to analyze these proteins for understanding SARS-CoV-1 and SARS-CoV-2. Mainly, our bioinformatic method provides a fast methodology to investigate all possible mutations, which can also predict the potential dominant variants of coronaviruses in future pandemics. Recently, several small inhibiting molecules have been designed to target the interaction between SARS-CoV-2 and hACE2 (Xiong et al., 2021; Yang et al., 2021). Key interface residues highlighted in our results can be good therapeutic targets.

## Conclusion

Saturated computational mutagenesis of SARS-CoV-1 S protein proved to be effective in analysing energy changes. Missense mutations in key residues such as A430 and S500 stabilized and destabilized SARS-CoV-1 full-length S and RBD, respectively. Moreover, missense mutations on residues G488 and T487 weakened the binding affinity of SARS-CoV1 S to hACE2. Mutation pathogenicity analysis showed that most highly destabilizing and highly stabilizing missense mutations would have a damaging effect on the SARS-CoV-1 S function. We also showed that missense mutations on N-linked glycosylation sites would destabilize SARS-CoV-1 S. The analysis of viral isolates from 2002 to 2003 and 2003–2004 showed that residue changes N479K, L472P, and F360S destabilized the S protein of 2003–2004 viral isolate leading to a reduction in infection rate. In addition, T487S and L472P weakened the binding affinity of SARS-CoV-1 S RBD. The comparison of different prediction tools showed consensus in predicting destabilizing mutations. Whereas the prediction of stabilizing mutations by the six prediction tools were inconclusive. Finally, most of the S missense mutations on SARS-CoV-1 had a similar stabilizing or destabilizing effect on corresponding residues on SARS-CoV-2. This approach can provide large-scale mutagenesis for future experimental studies on the coronavirus research.

## Data Availability

The original contributions presented in the study are included in the article/Supplementary Material, further inquiries can be directed to the corresponding authors.
